# H_2_ Gas Improves Functional Outcome After Cardiac Arrest to an Extent Comparable to Therapeutic Hypothermia in a Rat Model

**DOI:** 10.1161/JAHA.112.003459

**Published:** 2012-10-25

**Authors:** Kei Hayashida, Motoaki Sano, Naomi Kamimura, Takashi Yokota, Masaru Suzuki, Yuichiro Maekawa, Akio Kawamura, Takayuki Abe, Shigeo Ohta, Keiichi Fukuda, Shingo Hori

**Affiliations:** Department of Emergency and Critical Care Medicine, Keio University, Tokyo, Japan (K.H., M. Suzuki, S.H.); Department of Cardiology, Keio University, Tokyo, Japan (M. Sano, Y.M., A.K., K.F.); Department of Biochemistry and Cell Biology, Institute of Development and Aging Science, Graduate School of Medicine, Nippon Medical School, Kanagawa, Japan (T.A.); Center for Clinical Research, School of Medicine, Keio University, Tokyo, Japan (N.K., T.Y., S.O.)

**Keywords:** cardiac arrest, cardiopulmonary resuscitation, hydrogen gas, therapeutic hypothermia, ventricular fibrillation

## Abstract

**Background:**

All clinical and biological manifestations related to postcardiac arrest (CA) syndrome are attributed to ischemia–reperfusion injury in various organs including brain and heart. Molecular hydrogen (H_2_) has potential as a novel antioxidant. This study tested the hypothesis that inhalation of H_2_ gas starting at the beginning of cardiopulmonary resuscitation (CPR) could improve the outcome of CA.

**Methods and Results:**

Ventricular fibrillation was induced by transcutaneous electrical epicardial stimulation in rats. After 5 minutes of the subsequent CA, rats were randomly assigned to 1 of 4 experimental groups at the beginning of CPR: mechanical ventilation (MV) with 2% N_2_ and 98% O_2_ under normothermia (37°C), the control group; MV with 2% H_2_ and 98% O_2_ under normothermia; MV with 2% N_2_ and 98% O_2_ under therapeutic hypothermia (TH), 33°C; and MV with 2% H_2_ and 98% O_2_ under TH. Mixed gas inhalation and TH continued until 2 hours after the return of spontaneous circulation (ROSC). H_2_ gas inhalation yielded better improvement in survival and neurological deficit score (NDS) after ROSC to an extent comparable to TH. H_2_ gas inhalation, but not TH, prevented a rise in left ventricular end-diastolic pressure and increase in serum IL-6 level after ROSC. The salutary impact of H_2_ gas was at least partially attributed to the radical-scavenging effects of H_2_ gas, because both 8-OHdG- and 4-HNE-positive cardiomyocytes were markedly suppressed by H_2_ gas inhalation after ROSC.

**Conclusions:**

Inhalation of H_2_ gas is a favorable strategy to mitigate mortality and functional outcome of post-CA syndrome in a rat model, either alone or in combination with TH.

## Introduction

Despite advances in the management of patients who suffer a nontraumatic cardiac arrest (CA), survival rates remain low, and many survivors are left with neurological and cardiac sequelae.^1^ Post-CA syndrome, including neurological dysfunction, cardiac damage, and “sepsis-like” systemic inflammation, is likely to contribute to the multisystem organ dysfunction and ultimate demise of many CA victims.^2^ Therapeutic hypothermia (TH) is widely accepted as the gold-standard method to improve survival and limit neurological outcomes in patients who achieve return of spontaneous circulation (ROSC) after CA. Despite that, it is still underutilized.^3^ Thus, the development of alternative approaches with or without TH is an unmet medical need in ameliorating the prognosis of post-CA patients.

Molecular hydrogen (H_2_) has many potential therapeutic applications as a novel antioxidant.^4,5^ Since the first article reporting H_2_ effects, in *Nature Medicine* in 2007,^6^ the protective effects of H_2_ have been confirmed in different animal models, including limiting the infarct volume of brain^6^ and heart^7^ by reducing ischemia–reperfusion injury without altering hemodynamic parameters and providing protection against multiple-organ damage elicited by generalized inflammation.^8^ There are also some preliminary clinical data on this topic.^9–17^

All clinical and biological manifestations related to post-CA syndrome are attributed to ischemia–reperfusion injury in various organs including brain and heart. This study tested the hypothesis that inhalation of H_2_ gas during hyperoxic resuscitation can improve CA outcome. TH was chosen as the gold standard endorsed by professional societies and backed up by a significant body of evidence.^18–25^ We subjected rats to 5 minutes of ventricular fibrillation (VF) cardiac arrest (CA), followed by therapeutic hypothermia (TH), H_2_ treatment, or a combination of both. Controls were subjected to normothermic conditions. All groups were ventilated with 98% O_2_.

## Materials and Methods

### Animal Preparation

Fifteen-week-old male Wistar ST rats weighing an average of 373 g were used according to institutional approval by the Animal Ethics Committee. Rats were housed in a rodent facility under a 12-hour light–dark cycle during this study.

For experiments, rats were fasted overnight except for free access to water and then anesthetized with an intraperitoneal injection of pentobarbital sodium (45 mg/kg). The surgical procedures were carried out as previously described.^19,20^ The tracheas of the animals were intubated through a tracheostomy with a 14-gauge cannula and mechanically ventilated with a tidal volume (TV) of 0.65 mL/100 g, a respiratory rate (RR) of 100/min, and an FiO_2_ of 0.21 (Ventilator: SN-480-7, Shinano, Japan). Polyethylene catheters (PE50, Natsume, Japan) were inserted into the left femoral artery and vein and flushed intermittently with saline solution containing 2.5 IU/mL bovine heparin. Arterial blood pressure was measured, and an electrocardiogram was recorded by subcutaneous needle electrodes. Core temperature was monitored by a rectal temperature probe (BAT-10, Physitemp Instruments Inc) and maintained by a heating plate (SCP-85, AsOne, Japan) throughout the experiment to ensure appropriate temperature management.

### Ventricular Fibrillation and CPR

Ventricular fibrillation (VF) was induced by electrical stimulation via a transthoracic epicardium electrode, as previously described.^26^ The stimulator (Isostim, World Precision Instrument Inc) was used to perform direct and constant electrical stimulation of the epicardium with crude current, continuous single stimulation, a delay of 100 ms, a wave width of 1 ms, a frequency of 50 Hz, an intensity of 1 mA, and a stimulation duration of 3 minutes. Five minutes after initiation of VF, advanced cardiac life support was started; the rats were ventilated (0.65 mL/100 g, 100 breaths/min), and then chest compressions (200/min) were started by a finger of the same investigator using a metronome assistant. Adrenalin (2 μg/100 g) and 0.1 mL sodium bicarbonate (8.4%) were immediately administered to the rats at the beginning of CPR. Repeated doses were administered at 3-minute intervals as needed. Defibrillation (Nihon Koden, Tokyo, Japan) was performed with direct-current single-phase wave 3 J if the electrocardiogram displayed VF 1 minute after CPR. If the defibrillation failed, CPR was repeated, and defibrillations were again performed 1 minute after CPR. If the spontaneous circulation of the rats was not restored after 10 minutes with the above treatment, CPR was considered a failure.

After ROSC, rats were mechanically ventilated and invasively monitored for 2 hours in maintaining the target temperature. Rats were continuously given 1 mL/h isotonic saline for 2 hours after ROSC. After a recovery period of 2 hours, telemetry probes (Mini mitter, Respironics Inc) were implanted into the inguinal cavities to monitor activity. Rats were then weaned from the ventilator, extubated, and returned to their cages with easily accessible food and water. The survival time after CPR was recorded up to 72 hours.

### Experimental Protocol

**Figure 1.**
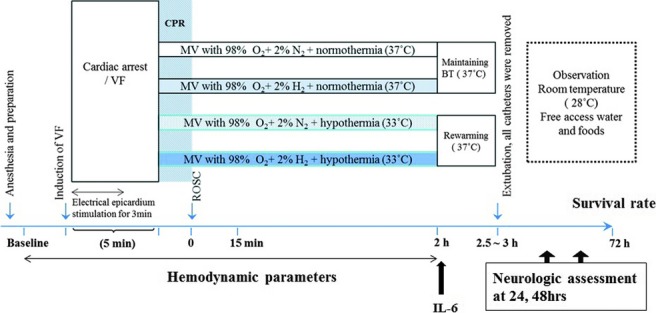
Experimental protocol of CPR and postresuscitation care in VF-induced cardiac arrest model. When mechanical ventilation was resumed (start CPR), rats were assigned to 1 of 4 experimental groups: mechanical ventilation (MV) with 98% O_2_+2% nitrogen (N_2_), the control group; MV with 98% O_2_+2% hydrogen (H_2_), the H_2_ group; MV with 98% O_2_+2% N_2_+therapeutic hypothermia (TH), the TH group; and MV with 98% O_2_+2% H_2_+TH, the H_2_+TH group. VF indicates ventricular fibrillation; MV, mechanical ventilation; BT, body temperature; and ROSC, return of spontaneous circulation.

Rats were randomly assigned to 1 of 4 experimental groups when mechanical ventilation (MV) was resumed at the beginning of CPR: MV with 2% N_2_ and 98% O_2_ at normothermia (the control group), MV with 2% H_2_ and 98% O_2_ at normothermia (H_2_ group), MV with 2% N_2_ and 98% O_2_ in targeting therapeutic hypothermia (the TH group), and MV with 2% H_2_ and 98% O_2_ in targeting TH (the H_2_+TH group) (Figure 1). The concentration of H_2_ in the gas mixture was determined using the Breath Gas Analyzer Model TGA-2000 (TERAMECS, Kyoto, Japan).

Baseline variables (weight, blood pressure, heart rate, body temperature, and preparation time) were the same in the 4 groups (Table 1).

**Table 1.** Baseline Physiological Variables

	Control (n=14)	H_2_ (n=14)	TH (n=14)	H_2_+TH (n=13)
Weight, g	373.6±6.1	367.5±7.9	376.4±8.6	375.9±4.8

Preparation time, minutes	42.4±1.2	41.7±0.8	40.1±0.8	40.1±1.2

HR at baseline, bpm	398±9	377±10	399±6	361±25

MBP at baseline, mm Hg	133±3	124±5	122±5	120±5

Temperature at baseline, °C	36.0±0.0	37.0±0.0	36.9±0.0	36.9±0.0

Values expressed as mean±SEM. TH indicates therapeutic hypothermia; HR, heart rate; MBP, mean blood pressure; CPR, cardiopulmonary resuscitation; ROSC, return of spontaneous circulation.

The concentration of 2% H_2_ was determined on the basis of previous observations^7^ as an optimal and safe concentration. Rats under ventilation received the respective gases via the tracheal tube until 2 hours after ROSC. In animals assigned to TH, body cooling was initiated coincident with the start of CPR. Rectal temperature was measured with a digital thermometer (BAT-10, Physitemp Instruments Inc) and taken as the body temperature, which was reduced to 33°C within 15 minutes with the aid of ice packs and a cooling plate (SCP-85, AsOne, Japan). Once reached, the target temperature was maintained for 2 hours after CPR and then returned to 37°C over a rewarming period of 30 to 60 minutes. For those animals not subjected to cooling, rectal temperature was maintained at 37°C using a hot plate (SCP-85, AsOne, Japan) for 1 hour after resuscitation. The analytical methods comprised 3 parts. In part 1, rats were divided randomly into 4 groups as described above; their neurological deficits were evaluated 24 and 48 hours after ROSC and their survival rate monitored up to 72 hours after ROSC. Arterial blood for blood gas analysis and peripheral venous blood was obtained at baseline and 10, 60, 90, and 120 minutes after ROSC. In part 2, myocardial functional recovery was monitored during the first 2 hours after ROSC, and then serum samples were obtained to measure cytokines level. In part 3, the sham-operated animals and the resuscitated animals, with or without H_2_ inhalation, were decapitated 24 hours after ROSC. The same surgical procedures, monitoring, and control of body temperature except for the induction of VF were done for the sham animals. Wet-to-dry weight ratios were measured for lung. Histopathological analyses of heart were performed to evaluate the favorable effect of H_2_ gas inhalation.

### Neurological Deficit Evaluation

Neurological deficit score (NDS) evaluations were performed by a single investigator who was unaware of group assignment. Consciousness and breathing, cranial nerve reflexes, motor function, sensory function, and coordination were scored according to an NDS system (0% to 100% scale; 0 normal, 100 brain death), as described previously.^27^

### Evaluation of Postresuscitation Myocardial Function

A Millar transducer catheter (SPR-320) was placed in the left ventricle (LV) cavity via the right internal carotid artery to monitor LV pressure using the polygraph system (Power Lab, ADInstrument, Castle Hill, Australia) before the induction of VF. LV systolic pressure (LVSP), LV end-diastolic pressure (LVEDP), and peak positive dP/dt and negative dP/dt were monitored during the first 2 hours after ROSC.

### Measurement of Serum IL-6 Level

Serum concentrations of IL-6 were measured 2 hours after ROSC in the 4 experimental groups and a sham-operated control group without VF with an ELISA Kit (OptEIA, BD Biosciences) according to the manufacturer's instructions.

### Lung Water Content Determined by Wet–Dry Method

Twenty-four hours after ROSC, the right lower lobes of the lungs were removed in a standard fashion, weighed immediately after removal, and then placed in a laboratory oven (60°C) for slow evaporation over 72 hours. The dried samples were weighed, and the water content (%) was calculated as (wet weight−dry weight)/(wet weight)×100%.

### Histopathological Analysis

Twenty-four hours after ROSC, the rats were decapitated. The hearts were quickly removed and fixed with Zamboni's solution. Coronal tissue slices (6-μm thickness) of the hearts (at the level of the left ventricle papillary muscle) were stained with hematoxylin and eosin and/or Azan–Mallory for histological evaluation.

For immunohistochemistry, the fixed sections were immunostained overnight at 4°C using a mouse monoclonal antibody against 4-hydroxy-2-nonenal (4-HNE; Japan Institute for the Control of Aging, NIKKEN SEIL Co, Ltd) to assess lipid peroxidation or a mouse monoclonal antibody against 8-hydroxy-deoxyguanosine (8-OHdG; Japan Institute for the Control of Aging, NIKKEN SEIL Co, Ltd) to detect the extent of nucleic acid oxidation.

In each Azan–Mallory-, 4-HNE-, and 8-OHdG-stained section, 4 slide fields were randomly examined using a defined rectangular field area (0.14 mm^2^). Images of Azan and 4-HNE staining were analyzed using Adobe Photoshop CS 5.1, and data of each staining section are reported as fibrotic tissue area stained blue (%) and 4-HNE relative intensity area (%) using automated counting software (Image J 1.46r, National Institute of Health). The 8-OHdG-positive cells were counted using automated counting software (Image J 1.46r, National Institute of Health), and the data were represented as the number of 8-OHdG-positive cells per field. A fluorescence microscope (Biorevo BZ-9000, Keyence, Osaka, Japan) was used for imaging.

### Statistical Analysis

Continuous variables are expressed as mean±SEM. Normally distributed data were analyzed by 1-way analysis of variance (ANOVA) with Bonferroni correction for post hoc comparisons between multiple experimental groups. NDSs were analyzed by Kruskal–Wallis with Mann–Whitney *U* analyses between multiple groups because the values are categorical variables. Kaplan–Meier analysis and the log-rank test were used to calculate survival rates. Hemodynamic and laboratory data were examined by a mixed-effects model for repeated-measures analyses, followed by ANOVA with Bonferroni correction for post hoc comparisons. The mixed-effects model for repeated-measures analysis contained treatment group, time, and treatment-by-time interaction as factors and random intercept for each subject. Significance was considered at the level of *P*<0.05. The Bonferroni-adjusted *P* value was defined such that the raw *P* value multiplies the number of comparisons. Statistical analyses were performed using SPSS software (SPSS Inc, Chicago, IL).

## Results

### Inhalation of H_2_ Gas Improved Early Post-ROSC Survival After Cardiac Arrest With Ventricular Fibrillation

First, we compared inhaled H_2_ gas with TH for the effect on early post-ROSC survival after VF-induced CA. Post-CA rats were assigned to the control group, H_2_ group, TH group, or H_2_+TH group at the time of CPR. There was no significant difference in terms of procedures for performing CPR, including CPR time to ROSC, dose of epinephrine, and number of defibrillations (Table 2). The resuscitation rates were 92.4% (13 survivors of 14 rats) in the control group, 92.4% (13 survivors of 14 rats) in the H_2_ group, 92.4% (13 survivors of 14 rats) in the TH group, and 100% (13 survivors of 13 rats) in the H_2_+TH group, respectively.

**Table 2.** Physiological Variables and Therapies During Cardiopulmonary Resuscitation

	Control (n=14)	H_2_ (n=14)	TH (n=14)	H_2_+TH (n=13)
CPR time to ROSC, seconds	117±20	99±18	109±11	106±18

Total dose of epinephrine, μg	8.5±1.0	8.0±0.8	8.0±0.0	8.5±0.5

Total administration of defibrillation	1.0±0.2	0.9±0.2	1.2±0.2	1.0±0.1

ROSC rate, n (%)	13 (92.9)	13 (92.9)	13 (92.9)	13 (100)

Temperature at the end of 2-hour protocol, °C	36.9±0.0	36.9±0.0	33.0±0.0*†	32.8±0.0*†

Values expressed as mean±SEM. TH indicates therapeutic hypothermia; CPR, cardiopulmonary resuscitation; ROSC, return of spontaneous circulation.

* *P*<0.001 compared with the control group.

† *P*<0.001 compared with the H_2_ group.

Arterial oxygen partial pressure (PaO_2_) was higher in the TH group compared with the controlled normothermia group, whereas serum potassium levels 60 and 120 minutes after ROSC were lower in the TH group compared with the controlled normothermia group. These results were consistent with evidence that hypothermia reduces oxygen consumption by 6% to 10% per degree Celsius^23^ and could potentially induce electrolyte loss.^23^ The groups did not differ in terms of partial pressure of carbon dioxide (PaCO_2_), pH, base excess, hematocrit, or lactate (Table 3).

**Table 3.** Group Arterial Blood Gas Analyses and Lactate Concentration Before Cardiac Arrest and in 2 Hours After Return of Spontaneous Circulation

		After ROSC		
		
	Baseline	10 Minutes	60 Minutes	120 Minutes
pH				

Control	7.48±0.03	7.18±0.02	7.37±0.02	7.36±0.03

H_2_	7.53±0.02	7.20±0.01	7.38±0.01	7.38±0.01

TH	7.51±0.01	7.19±0.01	7.35±0.01	7.31±0.01

H_2_+TH	7.53±0.01	7.18±0.01	7.36±0.00	7.34±0.00

PaO_2_, mm Hg**				

Control	86±3	314±51	438±35	480±19

H_2_	92±7	319±41	364±27	481±21

TH	84±3	513±38#†	575±19##††	582±21##††

H_2_+TH	92±4	433±41	534±22††	571±19#†

PaCO_2_, mm Hg				

Control	29.9±1.3	41.3±2.6	38.7±2.7	38.8±3.9

H_2_	28.9±1.7	42.5±1.8	36.9±2.2	38.1±1.9

TH	30.9±1.3	43.1±3.1	39.5±2.2	42.8±2.5

H_2_+TH	28.9±0.7	46.8±2.1	38.0±1.1	42.3±1.2

Na, mmol/L				

Control	139.8±0.5	140.7±1.0	140.3±0.6	139.5±0.5

H_2_	139.4±0.6	141.2±0.7	139.3±0.7	139.6±0.6

TH	140.8±0.6	141.3±0.7	140.2±0.8	141.3±0.7

H_2_+TH	139.6±0.4	142.8±0.6	141.9±0.8	140.2±0.7

K, mmol/L*				

Control	3.8±0.0	3.9±0.2	3.8±0.1	4.2±0.1

H_2_	3.9±0.0	3.8±0.2	3.8±0.1	4.2±0.1

TH	3.9±0.0	3.4±0.2	3.4±0.0#†	3.7±0.1#†

H_2_+TH	4.0±0.0	3.4±0.1	3.2±0.5#†	3.5±0.0#†

SaO_2_, %				

Control	97.5±0.3	99.0±0.6	99.9±0.0	100±0.0

H_2_	97.4±0.4	99.6±0.1	100±0.0	100±0.0

TH	97.2±0.3	99.9±0.0	100±0.0	100±0.0

H_2_+TH	97.6±0.4	99.9±0.0	100±0.0	100±0.0

Glucose, mg/dL**				

Control	196±8	238±22	293±15	269±20

H_2_	185±11	264±25	328±22	319±20

TH	193±16	258±24	339±21	338 ±38

H_2_+TH	178±10	205±18	304±10	337±17

Hematocrit, %				

Control	48.6±0.9	52.5±1.3	51.3±1.1	49.5±1.2

H_2_	48.1±1.1	54.0±0.9	50.3±0.8	49.0±0.8

TH	49.8±0.9	53.3±1.1	51.3±0.7	51.3±0.9

H_2_+TH	49.2±0.8	54.2±0.6	52.0±0.6	50.0±0.6

Lactate, mg/dL				

Control	1.4±0.1	8.9±0.7	2.3±0.3	2.0±0.2

H_2_	1.3±0.1	8.5±0.7	2.4±0.2	1.9±0.1

TH	1.7±0.2	8.9±0.7	3.1±0.6	2.8±0.9

H_2_+TH	1.4±0.1	8.4±0.4	3.0±0.2	1.9±0.2

Base excess, mmol/L				

Control	1.1±0.6	−12.7±1.2	−2.8±0.9	−3.5±0.9

H_2_	1.3±0.7	−11.1±0.8	−3.0±0.7	−3.1±0.8

TH	1.6±0.6	−11.7±1.0	−3.8±1.0	−4.1±1.4

H_2_+TH	1.6±0.6	−8.4±2.0	−3.7±0.7	−2.3±0.7

Values expressed as mean±SEM. Resuscitated animal were analyzed (n=13 for each group). TH indicates therapeutic hypothermia; ROSC, return of spontaneous circulation.

* *P*<0.05;

** *P*<0.01, statistically significant differences for treatment by time between 4 groups by mixed-effects model for repeated-measures analyses.

# *P<*0.05;

## *P<*0.01 vs the control group.

† *P<*0.05;

†† *P<*0.01 vs the H_2_ group.

**Figure 2.**
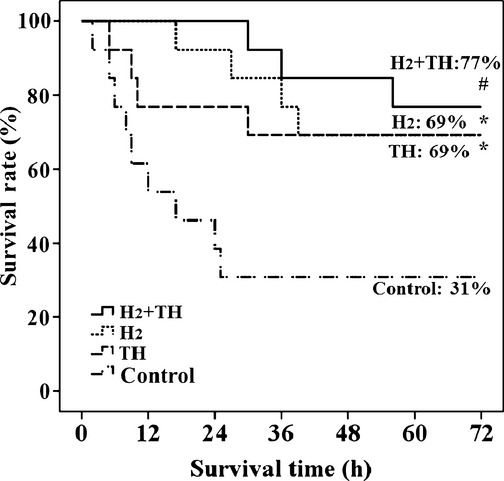
Kaplan–Meier analyses of cumulative survival at 72 hours. Statistically significant differences: ^#^*P*<0.01 compared with the control group; *P<0.05 compared with the control group. TH indicates therapeutic hypothermia.

The survival rate 24 hours after ROSC was 43% (6/13) in the control group, 92% (12/13) in the H_2_ group, 77% (10/13) in the TH group, and 100% (13/13) in the H_2_+TH group, whereas 72-hour survival rates were 31% (4/13), 69% (9/13), 69% (9/13), and 77% (10/13), respectively (Figure 2). These results indicated that inhalation of H_2_ gas during CPR and during the first 2 hours after ROSC significantly improved survival rate, comparable to those of hypothermia.

### Inhalation of H_2_ Gas Improved NDS in Postarrest State

**Figure 3.**
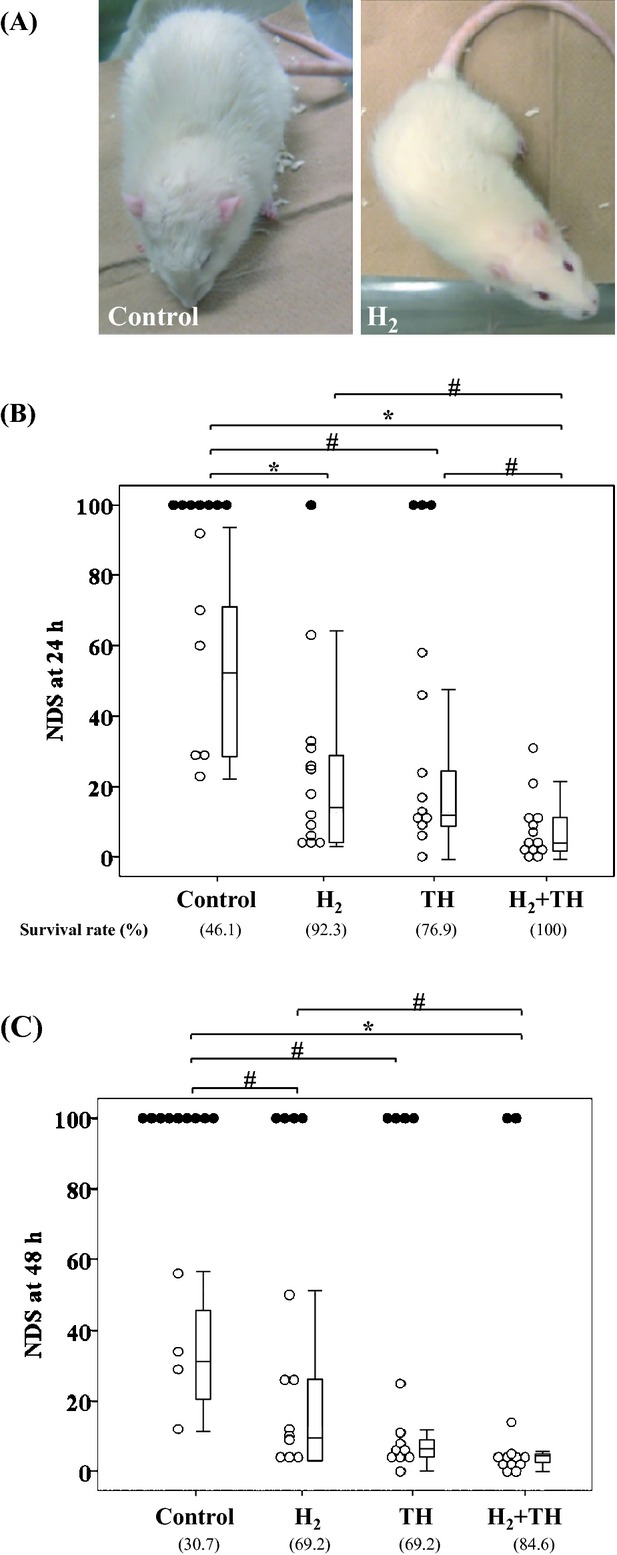
A, Representative photographs of rat appearance 24 hours after ROSC in the control group (left) and in the H_2_ group (right). Survival rate and neurological deficit scores (NDSs) 24 hours (B) and 48 hours (C) after ROSC. Dead rats (indicated by score=100) are indicated by closed circles. Box plots indicate NDS of survivors 24 hours and 48 hours after ROSC. TH indicates therapeutic hypothermia. Statistically significant differences: ^#^*P*<0.05 between groups; *P<0.001 between groups.

Interestingly, appearance was the most obvious difference between these groups of post-CA animals (Figure 3A). The typical sick-rat appearance of a hunched back and unkempt hair was often seen in post-CA control rats, whereas post-CA rats treated with H_2_ gas appeared healthy. All animals were evaluated for neurological function based on their NDS (100=worst NDS; 0=best NDS) 24 and 48 hours after ROSC. NDS 24 hours after ROSC was significantly lower in the H_2_ group (25.7±7.7%), TH group (38.0±10.7%), and H_2_+TH group (8.0±2.5%) compared with the control group (77.1±8.6%) (Figure 3B). The H_2_+TH group showed significantly better NDS 24 hours after ROSC compared with the H_2_ and TH groups. NDS 48 hours after ROSC was significantly better in the H_2_ group (41.9±11.7%), TH group (36.0±12.4%), and H_2_+TH group (18.5±10.0%) than in the control group (82.6±9.2%), with the H_2_+TH group showing a significantly better NDS 48 hours after ROSC than the H_2_ group (Figure 3C). These results indicated that functional outcome was improved by inhalation of H_2_ gas during CPR to an extent comparable to TH in post-CA rats. Inhalation of H_2_ gas plus TH had additive effect on NDS 24 hours after ROSC, but not 48 hours after ROSC, in the rat model of CA with ventricular fibrillation.

### Inhalation of H_2_ Gas Significantly Suppressed Elevations of IL-6 After Cardiac Arrest and Cardiopulmonary Resuscitation

Serum IL-6 levels 2 hours after ROSC markedly increased in post-CA rats of the controlled normothermia group (2239.9±440.8 pg/mL) compared with sham-operated rats (27.4±13.3 pg/mL), but were almost equal in post-CA rats of the TH (2431.2±634.7 pg/mL) and controlled normothermia groups, indicating that TH has little effect on the systemic inflammatory response at this point.

**Figure 4.**
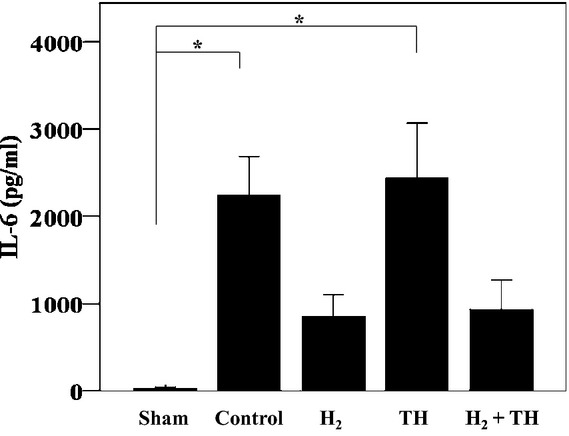
Serum IL-6 concentrations 2 hours after ROSC in the experimental and sham-operated groups. Bars represent the mean and standard error. *P<0.01 compared with the sham-operated group. ROSC indicates return of spontaneous circulation; TH, therapeutic hypothermia.

Serum IL-6 levels 2 hours after ROSC were not significantly increased in post-CA rats of the H_2_ group (848.9±257.0 pg/mL) or H_2_+TH group (933.3±339.0 pg/mL) compared with sham-operated rats, but in the H_2_+TH group, serum IL-6 levels were suppressed to the same degree as that of post-CA rats receiving only H_2_ gas. These results indicated that the elevation of IL-6 2 hours after ROSC was markedly suppressed by H_2_ gas inhalation (Figure 4).

### Inhalation of H_2_ Gas Kept LVEDP Low During the First 2 Hours After ROSC

We examined the impact of H_2_ gas inhalation on left ventricular functional recovery during the post-CA reperfusion period in comparison with TH. Hemodynamic parameters prior to induction of CA did not differ among the control, H_2_, TH, and H_2_+TH groups. Mean blood pressure was also not different among the 4 groups during the first 2 hours after ROSC; however, heart rate was significantly lower in the TH group compared with the control and H_2_ groups.

**Figure 5.**
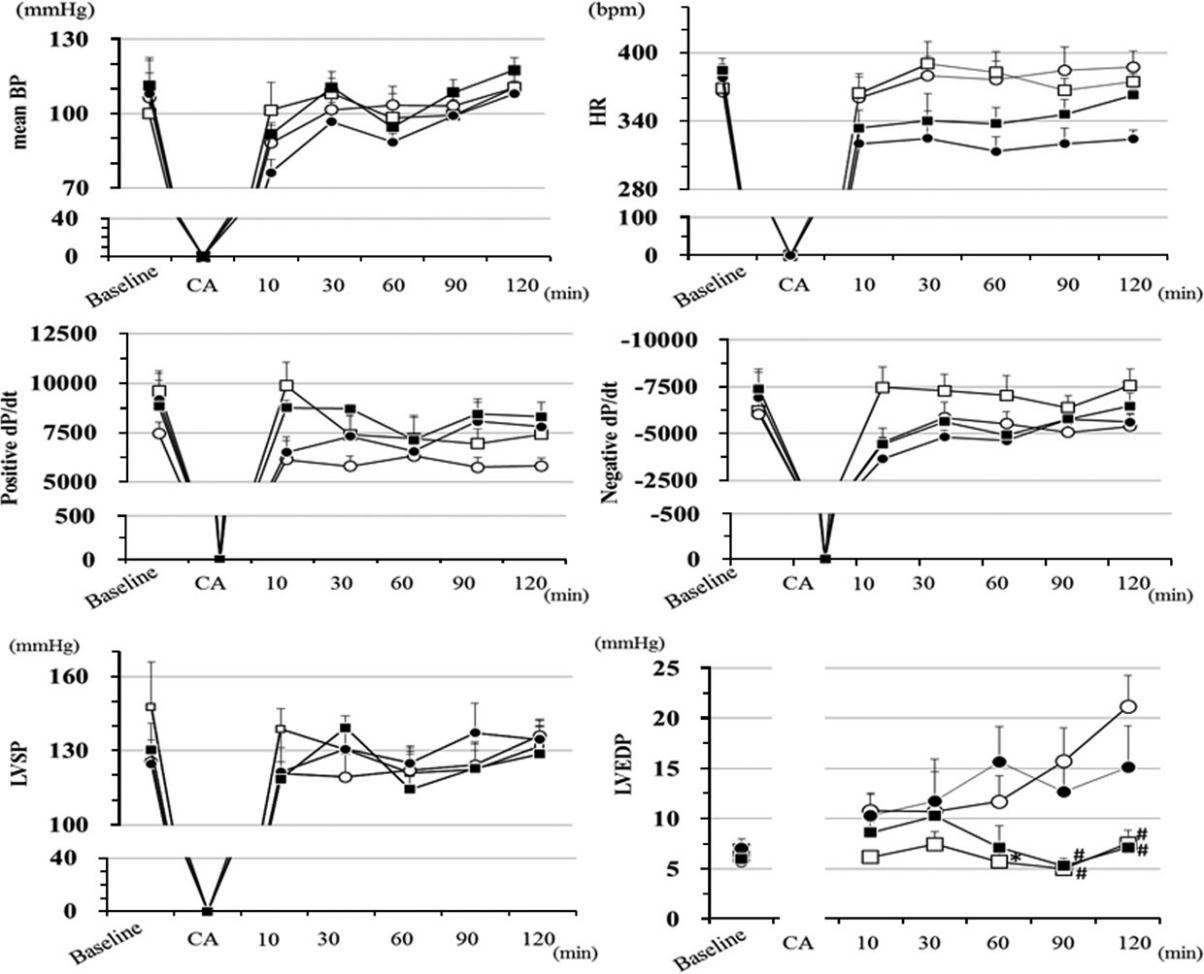
Changes in mean blood pressure (MBP), heart rate (HR), and LV peak positive, negative dP/dt and LV systolic pressure (LVSP), LV diastolic pressure (LVEDP), n=5 to 6; ^#^*P*<0.05 compared with the control group; *P<0.05 compared with the TH group.

Recovery of dP/dt max, an indicator of left ventricular systolic function, after 10 minutes of ROSC was significantly higher in the H_2_ and H_2_+TH groups than in the control and TH groups. Recovery of negative dP/dt max, an indicator of left ventricular diastolic function, after 10 minutes of ROSC was significantly superior in the H_2_ group compared with the other groups. This superiority conferred by H_2_ gas inhalation tended to continue during the first 2 hours after ROSC, and left ventricular systolic pressure was recovered after CA and ROSC to pre-CA levels. There was no difference in terms of left ventricular systolic pressure among the 4 groups during the first 2 hours after ROSC. By contrast, there was a striking difference with respect to left ventricular end-diastolic pressure (LVEDP), which gradually increased to ≥20 mm Hg 2 hours after ROSC in the control group. Remarkably, H_2_ gas inhalation kept LVEDP at pre-CA levels during the first 2 hours after ROSC (Figure 5), and this protective effect was observed both under controlled normothermia and under TH conditions.

### Inhaled Hydrogen Gas Attenuated Cardiomyocyte Degeneration and Necrosis, Inflammatory Cell Infiltration, Reactive Fibrosis, and Oxidative Stress in the Postcardiac Arrest Heart

**Figure 6.**
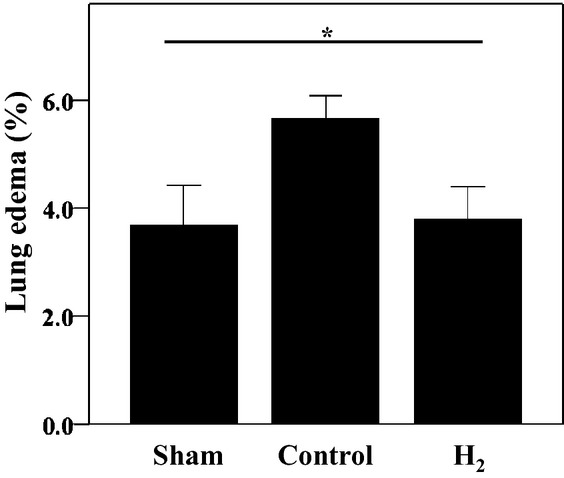
Effects of hydrogen on lung edema. Lung edema 24 hours after ROSC (n=3 to 6); *P<0.05 for analysis of variance between groups. Bars represent the standard error. ROSC indicates return of spontaneous circulation.

A favorable effect of inhaled H_2_ gas during CPR and during the first 2 hours after ROSC on functional recovery of the heart in rats after CA with VF prompted us to examine the pathohistological changes 24 hours after ROSC between control and H_2_ gas–treated rats. Consistent with the continuous elevation of LVEDP in post-CA rats of the control group, water content of the lung, an indicator of lung edema, tended to be higher in controls than in sham-operated rats, although lung water content was similar between post-CA H_2_ and sham rats (Figure 6).

**Figure 7.**
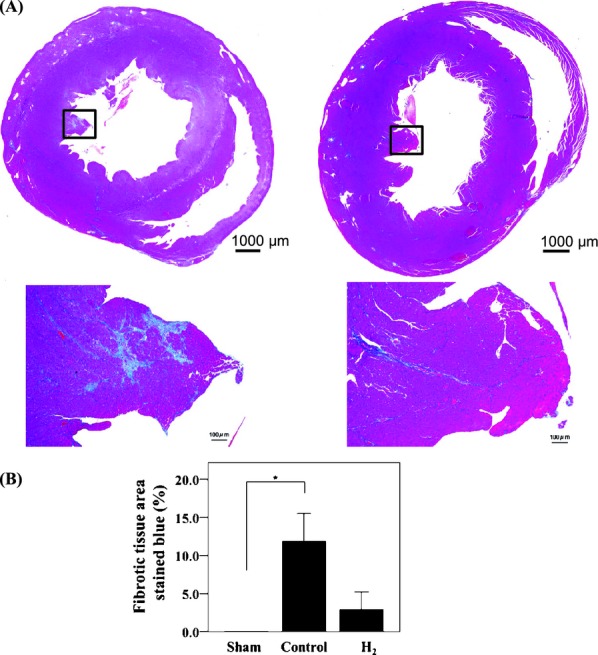
A, Representative photographs of Azan–Mallory staining of heart sections obtained 24 hours after ROSC in the absence (right) or presence (left) of H_2_ inhalation. B, Fibrotic tissue area stained blue (%) in sham and rats 24 hours after ROSC with or without H_2_ inhalation. Bars represent the standard error (n=4 to 6); *P<0.05 between groups. ROSC indicates return of spontaneous circulation.

Azan–Mallory-stained gross sections of whole heart also revealed that perivascular and interstitial fibrosis on the endocardial side of the myocardium emerged in the post-CA control rats 24 hours after ROSC but that this reactive fibrosis was less severe in the post-CA rats administered H_2_ gas (Figure 7).

**Figure 8.**
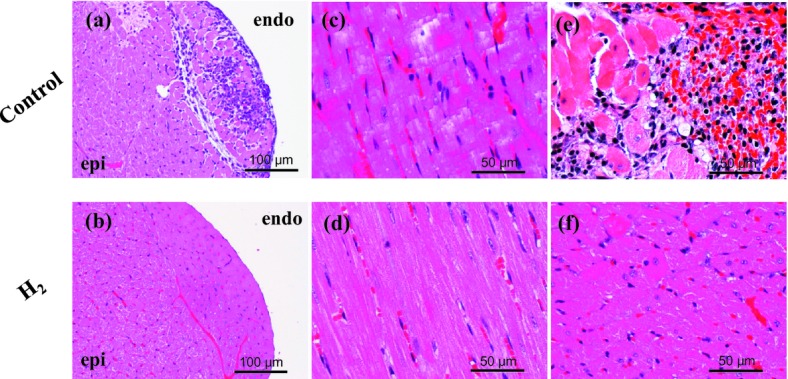
Hematoxylin and eosin staining of transverse sections of rat heart in the presence or absence of H_2_ inhalation. Shown is a microscopic view of inflammatory cell infiltration (a), contraction band necrosis (c), cytoplasmic eosinophilia (e), loss of nuclei (e), and vacuolar degeneration (e) in the left ventricle. These observations were reduced by inhalation of H_2_ (b, d, f).

Histological analysis with hematoxylin/eosin staining exhibited contraction band necrosis, coagulation necrosis with cytoplasmic eosinophilia, loss of nuclei, and vacuolar degeneration surrounded by inflammatory cell infiltration in the myocardium of the post-CA rats of the control group. These pathohistological changes were less severe in rats administered H_2_ gas (Figure 8).

**Figure 9.**
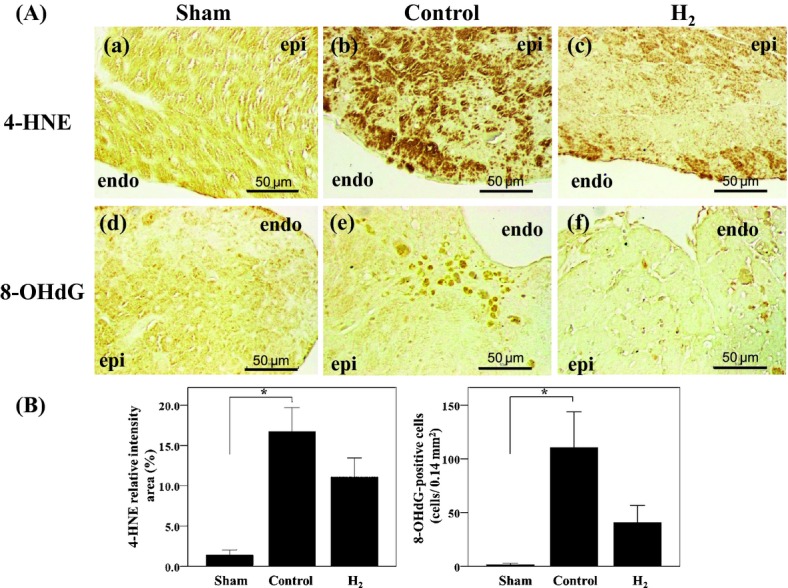
A, Immunohistochemical staining with antibodies against 4-HNE and 8-OHdG in transverse sections of sham and rats 24 hours after ROSC with or without H_2_ inhalation. H_2_ decreased levels of 4-HNE (b, c) and 8-OHdG (e, f). B, 4-HNE relative intensity area (%) and 8-OHdG-positive cells (cells/0.14) in sham and rats 24 hours after ROSC with or without H_2_ inhalation. Bars represent the standard error (n=4 to 6); *P<0.05 between groups.

Immunohistochemistry for 8-OH-dG (which is an index of oxidative DNA damage) and 4-HNE (which is the end product of lipid peroxidation) was carried out to investigate oxidative stress in myocardium obtained from the post-CA rats 24 hours after ROSC. The 8-OHdG-positive cells and 4-HNE-positive cardiomyocytes were distributed throughout the myocardium, particularly on the endocardial side of the myocardium. Notably, there were fewer 8-OHdG- positive cells and 4-HNE-positive cardiomyocytes obtained from the post-CA rats administered H_2_ gas (Figure 9). These results indicated that inhalation of H_2_ gas ameliorated oxidative myocardial injury during CPA and after ROSC.

## Discussion

Molecular hydrogen (H_2_) is a novel antioxidant with the following unique properties: (1) H_2_ is permeable to cell membranes and can target organelles, including mitochondria and nuclei; (2) H_2_ specifically quenches detrimental ROS such as •OH and peroxynitrite (ONOO−) while maintaining the metabolic oxidation–reduction reaction and other less potent ROS, such as O_2_^−^•, H_2_O_2_, and nitric oxide (NO•)^4,5^; and (3) inhaled H_2_ gas is rapidly transported and thus can reach “at-risk” ischemic organs including the brain in a timely fashion.^5^

We hypothesized that H_2_ therapy may improve outcomes of post-CA syndrome characterized by systemic ischemia–reperfusion injury and “sepsis-like” systemic inflammation because H_2_ treatment by oral, intravenous administration, or inhalation has demonstrated extended effectiveness in various situations, such as in brain ischemia–reperfusion injury,^6^ myocardial ischemia–reperfusion injury,^7^ sepsis,^28^ diabetes,^9,29^ intestinal grafts,^30^ hemodialysis,^11^ liver injury,^31^ and spinal cord injury^32^ and in animal models of Parkinson's disease^33^ and Alzheimer's disease.^34^ The present study demonstrated for the first time that inhalation of 2% H_2_ gas starting at the beginning of CPR and given for 2 hours after ROSC significantly improves the functional status of the brain (based on NDS) and heart and suppresses the systemic inflammatory response, thereby improving survival rate in a rat model of CA with VF. The salutary impact of H_2_ gas could be at least partially attributed to its radical-scavenging effect in the heart, because both 8-OHdG- and 4-HNE-positive cardiomyocytes obtained from the post-CA rats 24 hours after ROSC were markedly suppressed by H_2_ gas inhalation. The improved appearance in the H_2_ group relative to control animals could be at least in part a consequence of better cardiovascular performance, because lung edema tended to been diminished in the H_2_ group compared with the controls. Because the neurological assessment was based only on the NDS in this study, we do not have compelling evidence that the benefits were mediated via attenuation of brain injury in the present study.

We found that H_2_ inhalation had protective and, in some aspects, superior effects, comparable to those of hypothermia. The results are surprising, because TH is believed to confer protection against reperfusion injury by multiple mechanisms,^23^ including the suppression of free radicals, enzymes, and excitatory and inflammatory reactions, in addition to the direct physical protection of membranes, whereas the cornerstone of H_2_ therapy is selective ROS attenuation, which is only 1 facet of TH therapy. All groups were ventilated with 98% O_2_, and increased reperfusion damage due to hyperoxia could have improved the chance of detecting the H_2_ protective effect. If H_2_ protects only from the harmful effects of hyperoxia, then avoiding hyperoxia/targeting normoxia might have the same benefit. Slow rewarming is also considered important in avoiding harmful systemic responses, including vasodilation, hypotension, and rebound cerebral edema. The optimal rate of warming is not known, but the consensus is currently about 0.25°C to 0.5°C of warming per hour.^35^ Rewarming speed after hypothermia in a rodent model of CA was variable among different TH protocols in previous research.^19,24,36^ Here, we chose 60 minutes of rewarming based on the high metabolic rate of rats compared with humans. However, a rapid rewarming speed after hypothermia might reduce the protective effects of cooling. Consistent with previous observations,^37^ blood lactate increased while the base excess decreased in post-CA rats, peaking 10 minutes after ROSC and returning to near baseline levels 60 minutes after ROSC. Although the prognostic value of these measures^38^ after CA have been reported in clinical studies, we could not detect any difference in the time course of blood lactate levels and base excess after ROSC among the control, H_2_, TH, and H_2_+TH groups.

Finally, we observed that TH did not affect IL-6 levels 2 hours after ROSC, whereas inhaled H_2_ gas did, although we did not aim to refute the anti-inflammatory effect of TH. Rather, we should emphasize the anti-inflammatory effect of H_2_. Previous in vitro studies and observations in patients with traumatic brain injury also demonstrated the effects of hypothermia on IL-6 levels.^39–41^ In addition, H_2_ gas inhalation significantly improved the survival rate and organ damage of septic mice with cecal ligation and puncture, and that favorable effect was accompanied by a reduction in serum and tissue proinflammatory cytokine levels.^28^

## Conclusions

This study provided novel evidence that H_2_ inhalation administered at the beginning of CPR and continued for 2 hours after ROSC markedly improves NDS, myocardial outcome, and 72-hour survival rate in rats after CA to an extent comparable to TH.

### Limitation of the Study

We could not conclude that whether H_2_ inhalation has the same beneficial outcome as TH because of the small sample size and low statistical power. Because neurological outcome was assessed on the basis of the NDS only, further studies are clearly needed to determine whether H_2_ directly confers neuroprotection in post-CA status. Whether H_2_ gas must be applied at the beginning of CPR or if a delayed application could have similar effect was also not tested in this study, and indeed, hypothermia could be applied in a delayed fashion. The use of H_2_ remains in its infancy, and further studies are necessary to delineate the enigmatic effect of H_2_ beyond that of a simple antioxidant.
